# Variation in surgical treatment advice for women with stress urinary incontinence: a study using clinical case vignettes

**DOI:** 10.1007/s00192-020-04295-4

**Published:** 2020-04-06

**Authors:** Jil Billy Mamza, Rebecca Geary, Dina El-Hamamsy, Ipek Gurol, Jonathan Duckett, Tahir Mahmood, Ash Monga, Philip Toozs-Hobson, Andrew Wilson, Douglas Tincello, Jan Van der Meulen

**Affiliations:** 1 Department of Health Services Research and Policy, London School of Hygiene & Tropical Medicine, London, WC1H 9SH UK; 2 University Hospitals of Leicester NHS Trust, Southampton, UK; 3 Medway NHS Foundation Trust, Kent, UK; 4 NHS Fife Victoria Hospital, Scotland, UK; 5 University Hospital Southampton NHS Foundation Trust, Southampton, UK; 6 Birmingham Women’s and Children’s NHS Foundation Trust, Birmingham, UK; 7 Department of Health Sciences, University of Leicester, Leicester, UK

**Keywords:** Case vignettes, Decision-making, Female urinary incontinence, Surgical treatment

## Abstract

**Introduction:**

The aim of this study was to determine how recommendations of gynaecologists on surgical treatment for stress urinary incontinence (SUI) were influenced by patient characteristics.

**Methods:**

Two hundred forty-five gynaecologists in the UK fully responded to an online questionnaire including 18 vignettes describing 7 clinical characteristics of women with SUI (age, body mass index, SUI type, previous SUI surgery, frequency of leakage, bother, physical status). The gynaecologists scored recommendations for surgery ranging from 1 ‘certainly not’ to 5 ‘certainly yes’. Mean scores were used to calculate the relative impact (‘weight’) of each clinical characteristic. Latent class analysis was used to distinguish groups of gynaecologists with a particular practice style because they responded to the patient characteristics captured in the case vignettes in a similar way.

**Results:**

The gynaecologists’ overall average recommendation score was 2.9 (interquartile range 2 to 4). All patient characteristics significantly influenced the recommendation scores (*p* always < 0.001) but their impact was relatively small. SUI type was most important (weight 23%), followed by previous SUI surgery (weight 21%). Latent class analysis identified five groups of gynaecologists with practice styles that differed mainly with respect to their mean recommendation score, ranging from 1.3 to 4.0.

**Conclusions:**

Surgical treatment advice in response to case vignettes was only minimally influenced by patient characteristics. There were five groups of gynaecologists whose inclination to recommend surgical treatment varied. This suggests that there is lack of consensus on the role of surgery as a treatment for SUI. A considerable number of gynaecologists were reluctant to recommend surgery.

**Electronic supplementary material:**

The online version of this article (10.1007/s00192-020-04295-4) contains supplementary material.

## Introduction

Surgical treatments for stress urinary incontinence (SUI) are offered when conservative treatments are ineffective [1]. Over the last 10 years, there have been increasing concerns about the long-term risks of synthetic mid-urethral mesh sling insertion, by far the most commonly used surgical procedure treatment for SUI [2]. As a result, the frequency of mid-urethral mesh sling insertions has rapidly declined worldwide, with a reduction by about 50% between 2008 and 2017 in the UK [3], which highlights a major change in surgical practice.

The decision to recommend surgery is complex and many factors need to be considered, including the patient's past management, comorbidities and the impact that the SUI has on quality of life. Recent UK guidelines emphasize that treatment decisions need to focus on providing a woman with the best outcomes from her own individual perspectives [4].

To investigate in a systematic and quantitative way how all these factors influence the decision to recommend surgical treatment, we carried out a national survey of gynaecologists in the UK with a specialist interest in urogynecology. The survey included a series of written case simulations (‘clinical case vignettes’) that describe a number of patient characteristics, including the type of urinary incontinence (UI) and severity of the SUI. The gynaecologists were asked to indicate whether they would recommend immediate surgical treatment for each of the patients described in these vignettes.

Surveys based on clinical case vignettes are increasingly being used as a cost-effective approach to study how clinicians make decisions on the diagnosis and treatment of patients [5], for example, to study what factors influence decisions to use neuromodulation for patients with refractory idiopathic overactive bladder syndrome [6].

In the current study, we used an experimental approach which is sometimes referred to as ‘conjoint analysis’. This type of analysis is based on the notion that people make complex decisions by combining information from various sources (‘cues’) [7]. The importance (‘weight’) that a decision-maker places on each cue can be studied by measuring the joint effects of the cues on decisions made in response to a series of hypothetical decisions [8]. In this way, we could estimate the extent to which the treatment recommendations of the gynaecologists were influenced by the women’s clinical characteristics. We also used latent class analysis to explore to what extent we could classify the gynaecologists into mutually exclusive groups with different ‘practice styles’, based on the pattern of their treatment recommendations in response to the characteristics described in the case vignettes in a similar way [9].

## Materials and methods

### Survey

In 2017 we carried out an online survey of all members of the British Society of Urogynaecology and members of the Royal College of Obstetricians and Gynaecologists who had indicated they had an interest in urogynecology. We invited 1139 clinicians practicing in the UK, including senior trainees. These clinicians are practising gynaecologists and urogynecologists. All of them can be expected to be involved in making decisions about surgical treatment and most of them can be expected to perform surgery themselves. Data were collected with a web-based programme and a link to the survey was emailed to potential participants. Three reminder emails were sent in the 1-month period following the first email.

The survey consisted of an information screen providing a brief description of the project, questions on the surgeons’ characteristics, a page providing additional information and a number of clinical assumptions (see below) that the gynaecologists needed to make when responding to the clinical case vignettes (for the full survey, see Table S1 in Supporting Information).

### Development of the clinical case vignettes

A three-stage approach was followed to determine the specific patient characteristics and their relevant levels to be included in the clinical case vignettes. In the first stage (‘item identification’), a non-systematic literature search was carried out to identify patient characteristics associated with the short- and long-term outcome of SUI surgery. One of the authors (JM) independently identified potentially relevant preoperative patient characteristics. Another author (DE) reviewed the list of characteristics to ensure that terminologies were consistent with clinical practice in the UK. Four senior clinical experts (DT, JD, AM and PTH) were given the opportunity to add to this long list if they felt that important characteristics were missed.

In the second stage (‘item reduction’), seven potential characteristics were selected that were considered most important: age, body mass index (BMI), type of UI, defined as pure SUI, stress pre-dominant mixed urinary incontinence (MUI) and MUI, previous SUI surgery, leakage, bother and physical status. Also, the relevant levels for each of these characteristics were determined, aiming to create maximum difference between the levels for each characteristic while ensuring that the clinical profiles captured in the case vignettes were relevant and realistic (Table 1 and Box).

**Table 1:** Patient characteristics and levels captured in the clinical case vignettes

Characteristics	Level
Age group, years	55
	68
	79
BMI (kg/m^2^)	23
	30
	36
Type of stress urinary incontinence	Pure stress urinary incontinence
	Stress-predominant mixed urinary incontinence
	Mixed urinary incontinence
Previous SUI surgery	None
	Mid-urethral tape (any route)
	Bladder neck injection
Frequency of leakage	About 2 or 3 times a week
	About once a day
	Several times a day
Bother	A bit of a problem
	Quite a problem
	A serious problem
Physical status	ASA grade 2
	ASA grade 3

BMI: body mass index; ASA: American Society of Anesthesiologists

Box: Example of a clinical case vignette

**Table:** 

***A 55-year****old woman presents with symptoms of****mixed incontinence****.* *She leaks****several times a day****. She says that her UI condition is affecting.* *her daily activities and is****a serious problem****for her. Her BMI is****36 kg/m***^***2***^*.* *Previous gynaecological history includes****mid-urethral tape****. She is****ASA grade 2****.* *Would you recommend that this patient has surgical treatment now?* *□ Certainly yes* *□ Probably yes* *□ Not sure* *□ Probably not* *□ Certainly not* ***PLEASE ASSUME THAT:*** ***PATIENTS*** *• have been referred by their GP for further assessment* *• have completed all conservative and behavioural treatments (e.g. frequency volume charts, pelvic floor exercises, etc.) without benefit* ***RESULTS OF EXAMINATION INDICATE*** *• abdominal examination - normal* *• midstream urinalysis results - all negative* *• post-void residual volume < 100mls* ***TYPES OF URINARY INCONTINENCE.*** *This survey focuses on the following conditions:* *• stress urinary incontinence* *• stress-predominant mixed urinary incontinence* *• mixed urinary incontinence (urodynamic stress incontinence with detrusor overactivity)* ***PHYSICAL STATUS.*** *We describe physical status by the American Society of Anaesthesiologists (ASA grade) classification.* ***Examples of patients with ASA grade 2*** *• hypertension: well controlled with one type of antihypertensive medication* *• diabetes: well controlled with oral medication or insulin, without diabetic complication* *• COPD/asthma: with productive cough and wheeze, well controlled by inhalers with rare episode of acute chest infection, not limiting lifestyle* ***Examples of patients with ASA grade 3*** *• hypertension: requiring multiple antihypertensive medications or not well controlled* *• diabetes: diabetic complications or not well controlled with oral medication or insulin* *• COPD/asthma: not well controlled, limiting lifestyle, with high dose of inhaler or oral steroids, with frequent episodes of acute chest infections*	

The total number of possible different cases that can be generated with the seven identified patient characteristics is 1458 (= 3^6^ × 2^1^) according to a full factorial design. In the third stage (‘experimental design’), we used an orthogonal fractional factorial design to reduce the number of clinical case vignettes to 18 using SPSS statistical software [10]. This type of design reduces the number of included vignettes, while maximizing the amount of information collected and retaining the absence of correlation between the patient characteristics [11].

The clinical case vignettes describe the clinical profile of women referred to secondary care for further assessment and management of their SUI. Each case profile consisted of a very short patient description according to the seven characteristics followed by one question: ‘Would you recommend that this patient has surgical treatment now?’ and the gynaecologists could score their recommendation on a 5-point Likert-type scale ranging from ‘certainly yes’ to ‘certainly not’.

### Clinical assumptions

When responding to the clinical case vignettes, the gynaecologists were asked to make two clinical assumptions. First, they had to assume that the women had been referred to secondary care by their general practitioner and had completed all conservative and behavioural treatments without benefit. The second assumption was that the abdominal examination was normal, the midstream urinalysis results were all negative, and post-void residual volume was < 100 ml.

### Statistical analyses

The characteristics of the participating gynaecologists were summarized using frequency distributions. We used the means of the response scores and the 25th and 75th percentiles to describe the recommendations of the gynaecologists for each of 18 clinical case vignettes.

To assess the relative influence of the clinical characteristics (‘weight’) on the gynaecologists’ recommendations, we calculated the means of the recommendation score according to the levels of each of the clinical characteristics. The weight of a clinical characteristic was defined as the difference between the lowest and the highest mean recommendation score for that characteristic, divided by the sum of these differences for all seven clinical characteristics [8]. In other words, these weights express as a percentage the influence of each of the seven characteristics on the gynaecologists’ recommendations relative to the total overall weight of all characteristics.

We used a mixed-effects analysis of variance model to test the statistical significance of differences in the gynaecologists’ recommendation scores according to level of the patient characteristics, taking into account that the recommendation scores for the 18 clinical case vignettes were nested within gynaecologists. The impact of the gynaecologists’ background characteristics (main specialty, gender and age) on their recommendations was analysed by testing the interaction between the patient characteristics and gynaecologists’ background characteristics with a likelihood ratio test for composite models. We treated the recommendation score as a continuous variable and used parametric statistical methods given that it has been demonstrated that these methods have maximum statistical power and are appropriate for analysis of Likert-type data [12].

Latent class analysis is a statistical approach that classifies individuals into mutually exclusive and exhaustive classes based on the pattern of their responses [9]. This statistical approach was used to determine if groups of gynaecologists could be identified whose treatment recommendation scores for each of the 18 case vignettes suggest that they respond to the patient characteristics captured in these case vignettes in a similar way or, in other words, that they have a similar practice style.

Gynaecologists who were classed within the same group are expected to be more homogeneous with respect to their recommendation scores than gynaecologists classed in different groups. The Aikaike information criteria (AIC) and the Bayesian information criteria (BIC) were used to determine the optimal number of latent classes.

The predicted posterior probabilities of latent class membership were used to assign each gynaecologist in a group. In case a gynaecologist did not have a posterior probability > 50% for one particular group, group membership was considered to be unknown.

All statistical analyses were performed using Stata 15 [13].

The project was funded by the National Institute for Health Research (NIHR) Health Services and Delivery Research (HS&DR) Programme (14/70/162) in response to a peer-reviewed application. Two lay members of the project advisory team advised on the conduct of the the study design and the interpretation of results. The funder had no role in the design and conduct of the study; collection, management, analysis and interpretation of the data; preparation, review or approval of the manuscript; and decision to submit the manuscript for publication.

## Results

Three hundred thirty-four gynaecologists participated in the survey, of which 245 (73.4%) fully completed the questionnaire (Supporting Information, Fig. S1). Of the 245 gynaecologists, 56.3% were male, while 55.9% indicated urogynecology as their main specialty area (Table 2).

**Table 2:** Characteristics of gynaecologists and mean recommendation scores

Characteristics	Number	%	Mean recommendation score (standard deviation)^*^
Total	245	100	2.87 (1.28)
Gender			
Female	106	43	2.88 (1.26)
Male	139	57	2.86 (1.30)
Age categories			
< 45 years	85	35	2.88 (1.27)
45–54 years	84	34	2.87 (1.30)
≥ 55 years	76	31	2.85 (1.27)
Clinical specialty			
Gynaecology	108	44	2.83 (1.27)
Urogynecology	137	56	2.90 (1.29)
Trainee			
No	212	87	2.86 (1.29)
Yes	33	13	2.91 (1.24)
Region of practice in the UK			
England	212	87	2.86 (1.28)
Northern Ireland	11	4	2.75 (1.13)
Scotland	15	6	3.03 (1.34)
Wales	7	3	2.90 (1.34)

*Mean score on a 5-point scale ranging from 1 = “certainly not” to 5 = “certainly yes”

### Recommendations for surgery

**Fig. 1:**
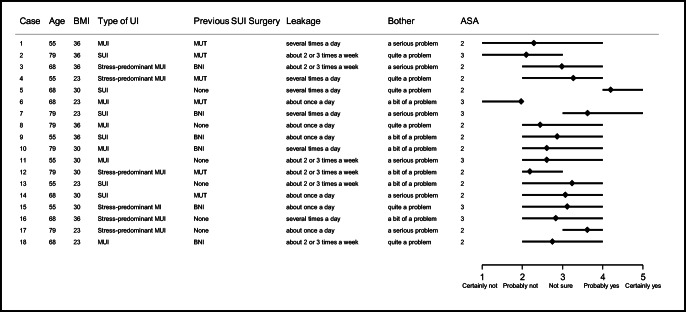
Surgical treatment recommendations of the gynaecologists for 18 clinical case vignettes. The range plots (horizontal bars) represent the 25th and 75th percentile and the mean of the recommendation score. SUI, stress urinary incontinence; MUI, mixed urinary incontinence; BNI, bladder neck injection; MUT, mid-urethral tape; BMI, body mass index in kg/m^2^; ASA, American Society of Anesthesiologists

Figure 1 shows the extent of variation in the recommendation scores of all gynaecologists for the 18 case profiles. The interquartile range was between 2 and 4 on the 5-point scale for 11 of the 18 vignettes. The scores were most strongly in favour of recommending surgery for case 5 (a 68-year-old woman with BMI of 30, with pure SUI, without previous SUI surgery, with leakage several times a day, for whom the incontinence is quite a problem and with an ASA of 2) and most strongly against recommending surgery for case 6 (a 68-year-old woman with BMI of 23, with MUI, with previous SUI surgery, with leakage one a day, for whom the incontinence is a bit of a problem and with ASA of 3).

### Impact of women’s characteristics on gynaecologists’ recommendations

**Fig. 2:**
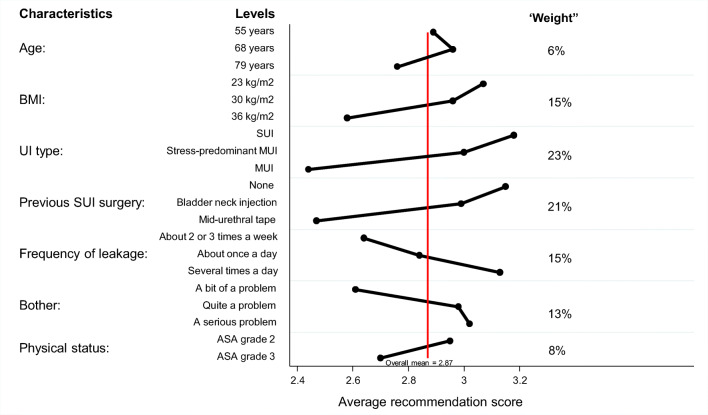
Influence of patient characteristics on gynaecologists’ recommendations for surgical treatment on a 5-point scale ranging from 1 ‘certainly not’ to 5 ‘certainly yes’. SUI, stress urinary incontinence; MUI, mixed urinary incontinence; BMI, body mass index in kg/m^2^); ASA, American Society of Anesthesiologists

Figure 2 demonstrates the impact of the patient characteristic on the gynaecologists’ recommendations. Overall, the impact of the patient characteristics was relatively small. The nature of the urinary incontinence had the greatest impact with a mean difference in the recommendation score for women with SUI and MUI of 0.8, which corresponds to a weight of 23% (see Methods), closely followed by the impact of a woman’s previous history of SUI surgery (weight of 21%) and—albeit it to a lesser extent—the frequency of urinary leakage (weight of 15%), BMI status (weight of 15%) and bother (weight of 13%). A woman’s physical status (weight of 8%) and her age (weight of 6%) had the least influence on the gynaecologists’ recommendations. The results of the mixed-effects analysis of variance indicated that all clinical characteristics captured in the case vignettes significantly influenced the recommendation score (*p* always < 0.001).

### Impact of gynaecologists’ characteristics on their recommendations

The average recommendation scores did not differ significantly according to the gynaecologists’ gender, age, specialty and trainee status (Table 2). In addition, there were no statistically significant differences between the scores of gynaecologists based in England, Scotland, Wales and Northern Ireland.

We did not find any evidence that the weights or relative influence of the patient characteristics on the recommendation scores varied according to the gynaecologists’ background characteristics on their recommendations (*p* value of interaction test for specialty, gender and age always > 0.05).

Additional analyses found that six gynaecologists consistently gave a recommendation score of 1 (‘certainly not’) to all 18 clinical case vignettes. Excluding the responses of these six gynaecologists had only a slight impact on the mean recommendation scores or the weights of the patient characteristics.

### Gynaecologists’ practice styles

**Fig. 3:**
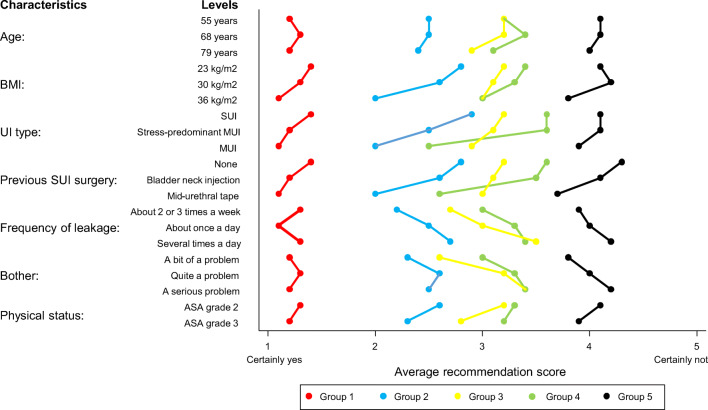
Influence of patient characteristics on gynaecologists’ recommendations for surgical treatment on a 5-point scale according to practice style group. SUI, stress urinary incontinence; MUI, mixed urinary incontinence; BMI, body mass index in kg/m^2^; ASA, American Society of Anesthesiologists

The results of the latent class analysis indicated that five mutually exclusive groups of gynaecologists could be identified with a different practice style (Fig. 3). The AIC and BIC both continued to go down as more latent classes were added, but there was a levelling off beyond a solution with five latent classes. Two hundred forty-four of the 245 respondents could be assigned to a particular group (see Methods).

The mean recommendation scores for the five practice style groups ranged from 1.25 to 4.04 (*p* < 0.001), which demonstrates a wide variation in the gynaecologists’ average inclination to recommend surgical treatment (Table 3). There were no significant differences in the gynaecologists’ characteristics between the groups.

**Table 3:** Characteristics of gynaecologists according to their practice style group derived from the latent class analysis (numbers and percentages unless otherwise indicated)

Characteristics	All	Group 1	Group 2	Group 3	Group 4	Group 5	P value
	244	17	85	52	69	21	
Mean recommendation score*	2.87	1.25	2.47	3.10	3.24	4.04	< 0.001
Gender							0.77
Female	105 (43.0%)	6 (35.3%)	41 (48.2%)	22 (42.3%)	27 (39.1%)	9 (42.9%)	
Age categories							0.60
< 45 years	71 (29.1%)	4 (23.5%)	28 (32.9%)	11 (21.2%)	23 (33.3%)	5 (23.8%)	
45–54 years	97 (39.8%)	10 (58.8%)	30 (35.3%)	24 (46.2%)	24 (34.8%)	9 (42.9%)	
≥ 55 years	76 (31.1%)	3 (17.7%)	27 (31.8%)	17 (32.7%)	22 (31.9%)	7 (33.3%)	
Clinical specialty							0.14
Gynaecology	107 (43.9%)	5 (29.4%)	43 (50.6%)	19 (36.5%)	34 (49.3%)	6 (28.6%)	
Urogynecology	137 (56.1%)	12 (70.6%)	42 (49.4%)	33 (63.5%)	35 (50.7%)	15 (71.4%)	
Trainee							0.55
Yes	33 (13.5%)	1 (88.2%)	10 (11.8%)	7 (13.5%)	13 (18.8%)	2 (9.5%)	

*Mean score on a 5-point scale ranging from 1 = “certainly not” to 5 = “certainly yes”

The impact of the women’s characteristics on gynaecologists’ recommendation scores is small compared with the differences in their average inclination to recommend surgical treatment among the five practice style groups. The impact of the women’s characteristics on the recommendation scores is relatively consistent (Fig. 3). The largest differences can be observed in the recommendations of the gynaecologists in group 2 and group 3, who seemed to give a greater weight to the BMI, type of UI and previous SUI surgery than the gynaecologists in the other groups.

## Discussion

### Main findings

Our nation-wide survey among UK gynaecologists with a special interest in urogynecology demonstrated wide variation in the recommendations given to a series of hypothetical patients. We could distinguish five groups of gynaecologists whose practice style differed widely with respect to their average inclination to recommend surgical treatment. In addition, we found that gynaecologists’ recommendations for surgical treatment were only minimally influenced by patient characteristics with type of UI and whether women had previously undergone surgical treatment having the greatest impact. Lastly, the gynaecologists’ recommendation scores did not seem to vary according to their gender, age, whether they indicated urogynecology as their sub-specialty status or their training status.

### Comparison with other studies

We observed that the impact of the patient characteristics was relatively small. An explanation for the slightly larger weights given to the type of UI and previous SUI surgery may be that these patient characteristics have been recognized as risk factors for a poor postoperative outcome [14]. This observation is also in line with results from a study, based on a survey of members of the International Urogynaecological Association, highlighting that clinical decisions in the management of recurrent SUI are guided by the type of previous surgery, urodynamic findings and individual surgeon’s preference [15].

The uniformity of the training of surgeons in the UK as well as the clinical governance structure in place in the UK’s National Health Service may explain why neither the surgeons’ demographic characteristics nor their main specialty or training status had an impact on how they responded to the patient characteristics represented in the clinical case vignettes. However, these results are in contrast with a number of recent clinician surveys. For example, a recent study involving general gynaecologists in the USA found their age to be a determinant of the surgical treatment options they offered [16]. Younger gynaecologists tended to have a smaller repertoire of surgical procedures than older gynaecologists. Similarly, a recent survey of UK specialists on how they managed recurrent or persistent SUI after the failure of primary surgery treatment demonstrated that training and the surgeons’ skills and experience played a vital role in their decision-making [17].

We observed that a woman’s age and BMI had relatively little impact on the gynaecologists’ recommendations for surgical treatment. This corresponds to an increasing body of evidence indicating that surgical treatments are effective in obese and in elderly patients [18–22]. On the other hand, it is also generally accepted that clinical decision-making is more complex given that overall health and functional status is poorer in these patient groups [21]. For example, it has recently been reported in a study of 12,000 women in the UK that an increased BMI is associated with poorer results of incontinence surgery according to outcomes reported by women themselves [23].

### Interpretation

Our study using case vignettes focused on the extent to which the gynaecologists’ recommendations for surgical treatment for women with SUI depend on patient characteristics. In clinical practice, however, women’s actual decisions about whether or not to undergo surgery will be informed by the recommendations from their gynaecologists as well as by their own preferences for treatment.

In the clinical case vignettes, we included patient characteristics that can only be derived from patient-reported information (frequency of leakage and bother) and those that are derived from information determined by the clinicians (type and severity of UI, previous surgery). We found that the weights assigned to the latter two (the clinician-derived characteristics) had a stronger combined influence on the gynaecologists’ recommendations than the patient-reported characteristics. It is important to note that the clinician-derived characteristics describe the severity and type of the urinary incontinence that have a recognized impact on the effectiveness of specific surgical treatments.

Most importantly, the results of our survey have to be interpreted in the light of the ongoing debate and increasing concerns about the safety of mid-urethral mesh tape procedures for women with SUI and the simultaneous rapid decline in the use of mid-urethral tape insertion as a treatment for SUI [2]. The large variation in the average inclination to recommend surgery among the five groups of gynaecologists that we could identify based on their practice style is likely to correspond to the substantial geographical variation that we demonstrated in the rate of SUI surgery in the English NHS between 2013 and 2016 [24]. This study, based on administrative hospital data, found a four-fold variation in the rate of surgery for SUI among 44 regional areas, each including on average about 500,000 adult women, set up to coordinate improvement of services in the English NHS.

Clinical uncertainty about the safety of using mid-urethral mesh tape insertions as well as fear of potential litigation is a likely explanation for both the variation in the average inclination among the five recognized practice style groups as well as the differences in rates of surgical treatment between the geographical areas [6]. The wide variation in practice style observed in our survey may also have medicolegal implications given that it can be argued that a clinician is not negligent if he or she acts in accordance with a practice accepted at the time as proper, even though some other clinicians adopt a different practice [25].

### Strengths and limitations

To our knowledge, our study is the second example of research using clinical case vignettes to investigate the extent to which specific patient characteristics influence gynaecologists’ treatment recommendations for women with urinary incontinence [6]. A strength of our study is that we invited all members of two professional bodies, practicing in the UK, who had indicated a specialist interest in urinary incontinence.

Of the 1139 gynaecologists who were invited to participate, only 245 fully responded. An additional analysis, comparing the characteristics of the gynaecologists who fully completed the survey with the 334 who only submitted partially completed surveys, did not show statistically significant differences in their characteristics (gender, age, specialty and training status), which—albeit indirectly—provides some evidence for the representativeness of the gynaecologists that fully responded to our survey. It has to be noted, however, that clinicians whose main specialty is urology were not included and their practice and views may be different. As a result, our findings are only applicable to gynaecological practice.

The validity of conjoint analysis as an experimental approach to get a better understanding of how clinicians respond to clinical cues is well established [26, 27]. This technique is frequently used to elicit views of health practitioners on the desirability of particular treatment options [5, 28]. An important example supporting the validity of conjoint analysis is a study comparing decisions to recommend surgery made in response to clinical case vignettes—very similar to those used in the current study—and those made for actual patients with aortic stenosis [29], which showed strong agreement in the way that that clinicians responded to the clinical characteristics captured in the clinical case vignettes and those for actual patients.

The use of conjoint analysis required that the recommendations of the clinicians for each case vignette were captured on a unidimensional scale (recommendation for surgery ranging from ‘certainly yes’ to ‘certainly not’). As a result, we were not able to include different alternatives for surgical treatment in the vignettes. In the UK, these alternatives may include continence tampons but not pessaries as the latter are not included as a recommended treatment option for urinary incontinence in recent UK guidelines [1]. Our results clearly demonstrate however that in the UK a considerable number of gynaecologists with a specialist interest in urogynecology are reluctant to recommend surgical treatment for women with urinary incontinence who have completed all conservative and behavioural treatments without benefit.

## Conclusions

In the UK, surgical decision-making for female SUI was most strongly influenced by the type of UI and previous SUI surgery. We found five groups of gynaecologists with different practice styles, mainly related to their average inclination to recommend surgery. This demonstrated that there is a lack of consensus on the role of surgery as a treatment option for SUI, which may correspond to the clinical uncertainty about the safety of incontinence surgery using mid-urethral mesh tape insertions.

## Electronic supplementary material

ESM 1 (DOCX 48 kb)
