# Supplementary material for: Variation in surgical treatment advice for women with stress urinary incontinence: a study using clinical case vignettes
Source: Int Urogynecol J. 2020 Apr 6;31(6):1153–61. doi: 10.1007/s00192-020-04295-4 (PMC7270981; doi:10.1007/s00192-020-04295-4)
Supplement: Supplementary file 1 — (DOCX 48 kb) [file 192_2020_4295_MOESM1_ESM.docx]

**VARIATION IN SURGICAL TREATMENT ADVICE FOR WOMEN WITH STRESS URINARY INCONTINENCE: A STUDY USING CASE VIGNETTES**

**SUPPORTING INFORMATION**

**List of Figures**

Figure S1: Sample selection process

**List of Tables**

Table S1: Full survey

**Figure S1: Sample selection process**

Survey sent to **1,139** clinicians via email

**334** **(29%)** responses returned

**245 (73%)** fully completed questionnaires

**Table S1: Pilot survey feedback questions**

| ASSUMPTIONS  Please assume that:  **PATIENTS**   - have been referred by their GP for further assessment - have completed all conservative and behavioural treatments (e.g. frequency volume charts, pelvic floor exercises, etc.) without benefit   **RESULTS OF EXAMINATION INDICATE**   - abdominal examination - normal - midstream urinalysis results - all negative - post-void residual volume < 100mls   ADDITIONAL INFORMATION  **CONDITION**  This survey focuses on the following UI conditions:   - Stress incontinence - Stress-predominant mixed incontinence - Mixed incontinence (urodynamic stress incontinence **with** detrusor overactivity)   **SURGICAL TREATMENT**  For each patient please indicate whether you would recommend surgical treatment.  Surgical treatment here refers to any surgical procedure you would consider appropriate.  **ASA GRADE**  We describe physical status by the American Society of Anaesthesiologists (ASA grade) classification.  ***Examples of patients with ASA grade 2***   - *Hypertension*: Well controlled with one type of antihypertensive medication - *Diabetes*: Well controlled with oral medication or insulin, without diabetic complication - *COPD / Asthma*: With productive cough and wheeze, well controlled by inhalers with rare episode of acute chest infection, not limiting lifestyle   ***Examples of patients with ASA grade 3***   - *Hypertension*: Requiring multiple antihypertensive medications, or not well controlled - *Diabetes*: Diabetic complications, or not well controlled with oral medication or insulin - *COPD / Asthma*: Not well controlled, limiting lifestyle, with high dose of inhaler or oral steroids, with frequent episodes of acute chest infections |
| --- |

**Case descriptions**

**18 case vignettes included in analysis and 2 vignettes used as ‘hold-out profiles’**

| \| **Patient 1**  A **55-year** old woman presents with symptoms of **mixed incontinence**. She leaks **several times a day**. She says that her UI condition is affecting her daily activities and is **a serious problem** for her. Her BMI is **36 kg/m^2^**. Previous gynaecological history includes **mid-urethral tape**. She is **ASA grade 2**.  **Question**  Would you recommend that this patient has surgical treatment now?   \|  \| Certainly yes \| \| --- \| --- \| \|  \| Probably yes \| \|  \| Not sure \| \|  \| Probably not \| \|  \| Certainly not \| \| \| --- \| --- \| --- \| --- \| --- \| --- \| --- \| --- \| --- \| --- \| --- \| | \| **Patient 2**  A **79-year** old woman presents with symptoms of **stress incontinence.** She leaks **about two or three times a week**. She says that her UI condition is affecting her daily activities and is **quite a problem** for her. Her BMI is **36 kg/m^2^**. Previous gynaecological history includes **mid-urethral tape**. She is **ASA grade 3**.  **Question**  Would you recommend that this patient has surgical treatment now?   \|  \| Certainly yes \| \| --- \| --- \| \|  \| Probably yes \| \|  \| Not sure \| \|  \| Probably not \| \|  \| Certainly not \| \| \| --- \| --- \| --- \| --- \| --- \| --- \| --- \| --- \| --- \| --- \| --- \| |
| --- | --- | --- | --- | --- | --- | --- | --- | --- | --- | --- | --- | --- | --- | --- | --- | --- | --- | --- | --- | --- | --- | --- | --- |
|  |  |
| \| **Patient 3**  A **68-year** old woman presents with symptoms of **mixed incontinence**. She leaks **about once a day**. She says that her UI condition is affecting her daily activities and is **a bit of a problem** for her. Her BMI is **30 kg/m^2^**. Previous gynaecological history includes **bladder neck injection**. She is **ASA grade 3**.  **Question**  Would you recommend that this patient has surgical treatment now?   \|  \| Certainly yes \| \| --- \| --- \| \|  \| Probably yes \| \|  \| Not sure \| \|  \| Probably not \| \|  \| Certainly not \| \| \| --- \| --- \| --- \| --- \| --- \| --- \| --- \| --- \| --- \| --- \| --- \| | \| **Patient 4**  A **68-year** old woman presents with symptoms of **stress-predominant incontinence.** She leaks **about two or three times a week**. She says that her UI condition is affecting her daily activities and is **a serious problem** for her. Her BMI is **36 kg/m^2^**. Previous gynaecological history includes **bladder neck injection**. She is **ASA grade 2**.  **Question**  Would you recommend that this patient has surgical treatment now?   \|  \| Certainly yes \| \| --- \| --- \| \|  \| Probably yes \| \|  \| Not sure \| \|  \| Probably not \| \|  \| Certainly not \| \| \| --- \| --- \| --- \| --- \| --- \| --- \| --- \| --- \| --- \| --- \| --- \| |
|  |  |
| \| **Patient 5**  A **55-year** old woman presents with symptoms of **stress-predominant incontinence.** She leaks **several times a day**. She says that her UI condition is affecting her daily activities and is **quite a problem** for her. Her BMI is **23 kg/m^2^**. Previous gynaecological history includes **mid-urethral tape**. She is **ASA grade 2**.  **Question**  Would you recommend that this patient has surgical treatment now?   \|  \| Certainly yes \| \| --- \| --- \| \|  \| Probably yes \| \|  \| Not sure \| \|  \| Probably not \| \|  \| Certainly not \| \| \| --- \| --- \| --- \| --- \| --- \| --- \| --- \| --- \| --- \| --- \| --- \| | \| **Patient 6**  A **68-year** old woman presents with symptoms of **stress incontinence.** She leaks **several times a day**. She says that her UI condition is affecting her daily activities and is **quite a problem** for her. Her BMI is **30 kg/m^2^**. She has **no other** gynaecological history of note. She is **ASA grade 2**.  **Question**  Would you recommend that this patient has surgical treatment now?   \|  \| Certainly yes \| \| --- \| --- \| \|  \| Probably yes \| \|  \| Not sure \| \|  \| Probably not \| \|  \| Certainly not \| \| \| --- \| --- \| --- \| --- \| --- \| --- \| --- \| --- \| --- \| --- \| --- \| |
|  |  |
| \| **Patient 7**  A **68-year** old woman presents with symptoms of **mixed incontinence**. She leaks **about once a day**. She says that her UI condition is affecting her daily activities and is **a bit of a problem** for her. Her BMI is **23 kg/m^2^**. Previous gynaecological history includes **mid-urethral tape**. She is **ASA grade 3**.  **Question**  Would you recommend that this patient has surgical treatment now?   \|  \| Certainly yes \| \| --- \| --- \| \|  \| Probably yes \| \|  \| Not sure \| \|  \| Probably not \| \|  \| Certainly not \| \| \| --- \| --- \| --- \| --- \| --- \| --- \| --- \| --- \| --- \| --- \| --- \| | \| **Patient 8**  A **79-year** old woman presents with symptoms of **stress incontinence.** She leaks **several times a day**. She says that her UI condition is affecting her daily activities and is **a serious problem** for her. Her BMI is **23 kg/m^2^**. Previous gynaecological history includes **bladder neck injection**. She is **ASA grade 3**.  **Question**  Would you recommend that this patient has surgical treatment now?   \|  \| Certainly yes \| \| --- \| --- \| \|  \| Probably yes \| \|  \| Not sure \| \|  \| Probably not \| \|  \| Certainly not \| \| \| --- \| --- \| --- \| --- \| --- \| --- \| --- \| --- \| --- \| --- \| --- \| |

| \| **Patient 9**  A **79-year** old woman presents with symptoms of **mixed incontinence**. She leaks **about once a day**. She says that her UI condition is affecting her daily activities and is **quite a problem** for her. Her BMI is **36 kg/m^2^**. She has **no other** gynaecological history of note. She is **ASA grade 2**.  **Question**  Would you recommend that this patient has surgical treatment now?   \|  \| Certainly yes \| \| --- \| --- \| \|  \| Probably yes \| \|  \| Not sure \| \|  \| Probably not \| \|  \| Certainly not \| \| \| --- \| --- \| --- \| --- \| --- \| --- \| --- \| --- \| --- \| --- \| --- \| | \| **Patient 10**  A **55-year** old woman presents with symptoms of **stress incontinence.** She leaks **about once a day**. She says that her UI condition is affecting her daily activities and is **a bit of a problem** for her. Her BMI is **36 kg/m^2^**. Previous gynaecological history includes **bladder neck injection**. She is **ASA grade 2**.  **Question**  Would you recommend that this patient has surgical treatment now?   \|  \| Certainly yes \| \| --- \| --- \| \|  \| Probably yes \| \|  \| Not sure \| \|  \| Probably not \| \|  \| Certainly not \| \| \| --- \| --- \| --- \| --- \| --- \| --- \| --- \| --- \| --- \| --- \| --- \| |
| --- | --- | --- | --- | --- | --- | --- | --- | --- | --- | --- | --- | --- | --- | --- | --- | --- | --- | --- | --- | --- | --- | --- | --- |
|  |  |
| \| **Patient 11**  A **79-year** old woman presents with symptoms of **mixed incontinence**. She leaks **several times a day**. She says that her UI condition is affecting her daily activities and is **a bit of a problem** for her. Her BMI is **30 kg/m^2^**. Previous gynaecological history includes **bladder neck injection**. She is **ASA grade 2**.  **Question**  Would you recommend that this patient has surgical treatment now?   \|  \| Certainly yes \| \| --- \| --- \| \|  \| Probably yes \| \|  \| Not sure \| \|  \| Probably not \| \|  \| Certainly not \| \| \| --- \| --- \| --- \| --- \| --- \| --- \| --- \| --- \| --- \| --- \| --- \| | \| **Patient 12**  A **55-year** old woman presents with symptoms of **mixed incontinence**. She leaks **about two or three times a week**. She says that her UI condition is affecting her daily activities and is **a serious problem** for her. Her BMI is **30 kg/m^2^**. She has **no other** gynaecological history of note. She is **ASA grade 3**.  **Question**  Would you recommend that this patient has surgical treatment now?   \|  \| Certainly yes \| \| --- \| --- \| \|  \| Probably yes \| \|  \| Not sure \| \|  \| Probably not \| \|  \| Certainly not \| \| \| --- \| --- \| --- \| --- \| --- \| --- \| --- \| --- \| --- \| --- \| --- \| |
|  |  |
| \| **Patient 13**  A **79-year** old woman presents with symptoms of **stress-predominant incontinence.** She leaks **about two or three times a week**. She says that her UI condition is affecting her daily activities and is **a bit of a problem** for her. Her BMI is **30 kg/m^2^**. Previous gynaecological history includes **mid-urethral tape**. She is **ASA grade 2**.  **Question**  Would you recommend that this patient has surgical treatment now?   \|  \| Certainly yes \| \| --- \| --- \| \|  \| Probably yes \| \|  \| Not sure \| \|  \| Probably not \| \|  \| Certainly not \| \| \| --- \| --- \| --- \| --- \| --- \| --- \| --- \| --- \| --- \| --- \| --- \| | \| **Patient 14**  A **55-year** old woman presents with symptoms of **stress incontinence.** She leaks **about two or three times a week**. She says that her UI condition is affecting her daily activities and is **a bit of a problem** for her. Her BMI is **23 kg/m^2^**. She has no other gynaecological history of note. She is **ASA grade 2**.  **Question**  Would you recommend that this patient has surgical treatment now?   \|  \| Certainly yes \| \| --- \| --- \| \|  \| Probably yes \| \|  \| Not sure \| \|  \| Probably not \| \|  \| Certainly not \| \| \| --- \| --- \| --- \| --- \| --- \| --- \| --- \| --- \| --- \| --- \| --- \| |
|  |  |
| \| **Patient 15**  A **68-year** old woman presents with symptoms of **stress incontinence.** She leaks **about once a day**. She says that her UI condition is affecting her daily activities and is **a serious problem** for her. Her BMI is **30 kg/m^2^**. Previous gynaecological history includes **mid-urethral tape**. She is **ASA grade 2**.  **Question**  Would you recommend that this patient has surgical treatment now?   \|  \| Certainly yes \| \| --- \| --- \| \|  \| Probably yes \| \|  \| Not sure \| \|  \| Probably not \| \|  \| Certainly not \| \| \| --- \| --- \| --- \| --- \| --- \| --- \| --- \| --- \| --- \| --- \| --- \| | \| **Patient 16**  A **55-year** old woman presents with symptoms of **stress-predominant incontinence.** She leaks **about once a day**. She says that her UI condition is affecting her daily activities and is **a serious problem** for her. Her BMI is **30 kg/m^2^**. Previous gynaecological history includes **bladder neck injection**. She is **ASA grade 3**.  **Question**  Would you recommend that this patient has surgical treatment now?   \|  \| Certainly yes \| \| --- \| --- \| \|  \| Probably yes \| \|  \| Not sure \| \|  \| Probably not \| \|  \| Certainly not \| \| \| --- \| --- \| --- \| --- \| --- \| --- \| --- \| --- \| --- \| --- \| --- \| |

| \| **Patient 17**  A **55-year** old woman presents with symptoms of **stress-predominant incontinence.** She leaks **about once a day**. She says that her UI condition is affecting her daily activities and is **quite a problem** for her. Her BMI is **30 kg/m^2^**. Previous gynaecological history includes **bladder neck injection**. She is **ASA grade 3**.  **Question**  Would you recommend that this patient has surgical treatment now?   \|  \| Certainly yes \| \| --- \| --- \| \|  \| Probably yes \| \|  \| Not sure \| \|  \| Probably not \| \|  \| Certainly not \| \| \| --- \| --- \| --- \| --- \| --- \| --- \| --- \| --- \| --- \| --- \| --- \| | \| **Patient 18**  A **68-year** old woman presents with symptoms of **stress-predominant incontinence.** She leaks **several times a day**. She says that her UI condition is affecting her daily activities and is **a bit of a problem** for her. Her BMI is **36 kg/m^2^**. She has **no other** gynaecological history of note. She is **ASA grade 3**.  **Question**  Would you recommend that this patient has surgical treatment now?   \|  \| Certainly yes \| \| --- \| --- \| \|  \| Probably yes \| \|  \| Not sure \| \|  \| Probably not \| \|  \| Certainly not \| \| \| --- \| --- \| --- \| --- \| --- \| --- \| --- \| --- \| --- \| --- \| --- \| |
| --- | --- | --- | --- | --- | --- | --- | --- | --- | --- | --- | --- | --- | --- | --- | --- | --- | --- | --- | --- | --- | --- | --- | --- |
|  |  |
| \| **Patient 19**  A **79-year** old woman presents with symptoms of **stress-predominant incontinence.** She leaks **about once a day**. She says that her UI condition is affecting her daily activities and is **a serious problem** for her. Her BMI is **23 kg/m^2^**. She has **no other** gynaecological history of note. She is **ASA grade 2**.  **Question**  Would you recommend that this patient has surgical treatment now?   \|  \| Certainly yes \| \| --- \| --- \| \|  \| Probably yes \| \|  \| Not sure \| \|  \| Probably not \| \|  \| Certainly not \| \| \| --- \| --- \| --- \| --- \| --- \| --- \| --- \| --- \| --- \| --- \| --- \| | \| **Patient 20**  A **68-year** old woman presents with symptoms of **mixed incontinence**. She leaks **about two or three times a week**. She says that her UI condition is affecting her daily activities and is **quite a problem** for her. Her BMI is **23 kg/m^2^**. Previous gynaecological history includes **bladder neck injection**. She is **ASA grade 2**.  **Question**  Would you recommend that this patient has surgical treatment now?   \|  \| Certainly yes \| \| --- \| --- \| \|  \| Probably yes \| \|  \| Not sure \| \|  \| Probably not \| \|  \| Certainly not \| \| \| --- \| --- \| --- \| --- \| --- \| --- \| --- \| --- \| --- \| --- \| --- \| |
|  |  |
